# NiO/Ga_2_O_3_ Heterojunction with Tunable Oxygen Vacancies for Efficient Self-Powered Solar-Blind UV Detection

**DOI:** 10.3390/ma19030530

**Published:** 2026-01-29

**Authors:** Luyu Liu, Kangxin Shen, Huimin Su, Jintao Xu, Jiajun Lin, Yaping Li, Shuguang Zhang, Linfeng Lan, Junbiao Peng

**Affiliations:** 1 State Key Laboratory of Luminescent Materials and Devices, South China University of Technology, Guangzhou 510640, China; 202420118759@mail.scut.edu.cn (L.L.); suhuiminss@163.com (H.S.);; 2 School of Materials Science and Engineering, South China University of Technology, Guangzhou 510640, China; 3 Guangdong Provincial Key Laboratory of In-Memory Computing Chips, School of Electronic and Computer Engineering, Peking University, Shenzhen 518055, China

**Keywords:** self-powered, ultraviolet photodetector, gallium oxide, heterojunction, magnetron sputtering

## Abstract

Solar-blind ultraviolet (UV) photodetectors based on wide-bandgap oxide semiconductors are highly desirable for environmental monitoring, flame sensing, and secure optical communication. Among them, Ga_2_O_3_ has attracted extensive attention due to its ultra-wide bandgap and intrinsic solar-blind response; however, its high dark current, weak built-in electric field, and defect-induced instability remain critical challenges, particularly for amorphous films prepared by scalable sputtering processes. Herein, a self-powered solar-blind UV photodetector based on a NiO/Ga_2_O_3_ heterojunction is demonstrated, in which the oxygen-vacancy concentration and band structure of sputtered Ga_2_O_3_ are systematically regulated by tailoring the Ar/O_2_ sputtering atmosphere. Combined X-ray photoelectron spectroscopy, UV photoelectron spectroscopy, and optical measurements reveal that the variation in oxygen-vacancy concentration simultaneously modulates the Fermi-level position, band-edge alignment, and built-in potential at the NiO/Ga_2_O_3_ interface. As a result, the optimized heterojunction device exhibits a low dark current, pronounced rectifying behavior, and efficient carrier separation under zero bias, enabling self-powered operation. The photodetector delivers a responsivity of 47 mA W^−1^, a detectivity of 7.52 × 10^11^ Jones, and a high rejection ratio exceeding 10^4^ between 254 and 365 nm. Furthermore, stable and high-contrast UV imaging is successfully demonstrated, highlighting the practical applicability of the device. This work provides an effective methodology for modulating defects and band structure in high-performance solar-blind UV photodetectors based on sputtered wide-bandgap oxide heterojunctions.

## 1. Introduction

Solar-blind ultraviolet (UV) photodetectors operating in the 200–280 nm spectral range have attracted considerable attention for applications in space communication, disaster early-warning, environmental monitoring, and military reconnaissance [1,2,3,4], owing to their intrinsically high signal-to-noise ratio under Earth’s background radiation [5,6]. Compared with visible and near-UV photodetectors, solar-blind devices are immune to solar interference and therefore enable reliable detection without additional optical filtering, which is highly desirable for low-power and high-selectivity sensing systems. Among various wide-bandgap semiconductor materials, gallium oxide (Ga_2_O_3_) is considered an ideal candidate for high-performance solar-blind UV detection due to its exceptionally wide direct bandgap (4.6–5.2 eV) [7,8,9], and intrinsic absorption edge near 253 nm, which enables a high rejection ratio between deep-UV (254 nm) and near-UV (365 nm) wavelengths [10,11,12]. While the broader landscape of Ga_2_O_3_-based solar-blind photodetectors has been comprehensively reviewed recently [4], the specific impact of oxygen-vacancy modulation on sputtered amorphous Ga_2_O_3_ remains insufficiently understood. In particular, the correlation between sputtering atmospheres, defect chemistry, and NiO/Ga_2_O_3_ interfacial energies has not been systematically explored. This work focuses on how the Ar/O_2_ ratio regulates oxygen-vacancy concentration and band alignment, directly influencing the performance of self-powered solar-blind photodetectors.

Over the past decade, Ga_2_O_3_-based photodetectors have been extensively studied using a variety of growth methods [13,14,15], such as metal–organic chemical vapor deposition (MOCVD) [16], pulsed laser deposition (PLD) [17,18], and molecular beam epitaxy (MBE) [19]. For instance, Sn-doped Ga_2_O_3_ films were deposited on n-type Si substrates by MOCVD [20], while Ga_2_O_3_/SnO_2_/SnS heterojunctions were fabricated on sapphire via PLD [21]. In addition, highly efficient self-powered Ga_2_O_3_/Bi_2_Se_3_ heterojunction photodetectors were achieved by sequential MBE growth of Ga_2_O_3_ and Bi_2_Se_3_ layers [22]. While these approaches often rely on single-crystalline β-Ga_2_O_3_ films with excellent material quality, they typically involve complex fabrication processes, high cost, and stringent growth conditions, which limit scalability and practical applications [23]. In contrast, amorphous or nanocrystalline Ga_2_O_3_ thin films prepared by magnetron sputtering offer clear advantages in terms of low-temperature processing, large-area uniformity, compatibility with industrial platforms, and flexible tunability of electronic properties [24]. However, sputtered Ga_2_O_3_ films inevitably contain a high density of oxygen-related defects, which strongly influence their electronic structure and device performance. To overcome the limitations associated with high dark current and inefficient carrier separation in Ga_2_O_3_-based photodetectors, heterojunction device architectures have been widely adopted. Compared with photoconductive devices, heterojunction photodiodes can utilize the built-in electric field at the interface to enable efficient separation and transport of photogenerated carriers [25], thereby achieving faster response, lower dark current, and improved operational stability [26,27]. Considering the persistent difficulty of realizing reliable p-type doping in Ga_2_O_3_, constructing heterojunctions with stable p-type oxide semiconductors provides a practical alternative. Nickel oxide (NiO), a p-type wide-bandgap semiconductor (*E_g_* ~3.7 eV) with good transparency and chemical stability, has been demonstrated to be an effective counterpart for Ga_2_O_3_, forming type-II band alignment and enabling self-powered photodetection [28]. Nevertheless, the interfacial band alignment and built-in potential of NiO/Ga_2_O_3_ heterojunctions are highly sensitive to the defect chemistry and electronic structure of the Ga_2_O_3_ layer.

Despite considerable progress, most previous studies on sputtered Ga_2_O_3_ photodetectors have focused on tuning sputtering power, post-annealing treatments, or crystallization behavior to improve device performance [29,30,31]. In comparison, the direct regulation of oxygen vacancy concentration through sputtering atmosphere control and its impact on the absolute band positions and heterojunction energetics, has received far less systematic attention. In particular, a clear understanding of how the controlled modulation of oxygen-vacancy concentration in amorphous Ga_2_O_3_ simultaneously affects defect states, band structure evolution, and interfacial energy alignment in NiO/Ga_2_O_3_ heterojunctions remains lacking. This knowledge gap hampers the rational design of high-performance, self-powered solar-blind photodetectors based on scalable oxide thin films.

In this work, a self-powered solar-blind UV photodetector based on a NiO/Ga_2_O_3_ vertical heterojunction is developed by systematically varying oxygen vacancies in sputtered Ga_2_O_3_ films through precise control of the Ar/O_2_ ratio during magnetron sputtering. By continuously varying the deposition atmosphere from oxygen-deficient to near-stoichiometric conditions, the chemical composition and defect states of amorphous Ga_2_O_3_ are finely regulated, enabling controlled modulation of the Fermi-level position, band-edge alignment, and built-in potential at the NiO/Ga_2_O_3_ interface. As a result, the optimized heterojunction device operates efficiently in photovoltaic mode under zero bias, delivering a high responsivity of 47 mA W^−1^ at 254 nm, an excellent solar-blind rejection ratio (*R*_254_/*R*_365_ > 10^4^), and fast response dynamics with rise and fall times of 25 ms and 63 ms, respectively. These results establish a direct correlation between deposition parameters, defect chemistry, band alignment, and device performance, demonstrating that the controlled modulation of oxygen-vacancy concentration provides an effective and scalable strategy for optimizing heterojunction energetics and advancing high-performance solar-blind UV photodetectors.

## 2. Experimental Section

### 2.1. Materials

Heavily doped p-type silicon (p^+^-Si) substrates were purchased from Kaihua Shunchen Electronic Technology Co., Ltd. (Dongguan, China). NiO, Ga_2_O_3_, and ITO ceramic targets with a purity of 99.99% were purchased from NuoXing Advanced Material Co., Ltd. (Fuzhou, China). Silver (Ag) was used as the contact metal. Acetone and isopropyl alcohol were supplied by Guangzhou Shengying Chemical Co., Ltd. (Guangzhou, China). All materials were used as received without further purification.

### 2.2. Device Fabrication

**Figure 1:**
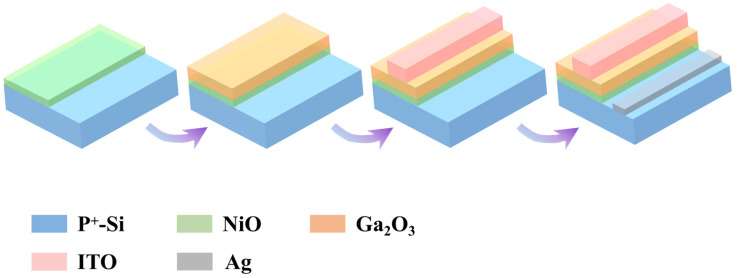
Schematic illustration of the sequential fabrication process for the NiO/Ga_2_O_3_ heterojunction UV photodetector.

A schematic illustration of the NiO/Ga_2_O_3_ heterojunction photodetector is presented in Figure 1. The p^+^-Si substrates were sequentially cleaned in isopropyl alcohol, deionized water, acetone, and isopropyl alcohol, with ultrasonication for 15 min in each solvent. After cleaning, the substrates were dried in an oven for 4 h. A 20 nm NiO layer was first deposited onto cleaned p^+^-Si substrates by radio-frequency (RF) magnetron sputtering using a ceramic NiO target. The deposition was performed at an RF power of 80 W, at a working pressure of 0.5 Pa, in an Ar atmosphere (Ar/O_2_ = 10/0) for 10 min, and with a sputtering duration of 10 min; these conditions were maintained for all NiO depositions in this work. Subsequently, a 50 nm Ga_2_O_3_ layer was deposited on top of the NiO by RF magnetron sputtering at 100 W and 0.5 Pa. To systematically investigate the effects of growth conditions, two sets of parameters were independently varied: (i) the annealing temperature, set to room temperature, 300 °C, and 500 °C and (ii) the sputtering atmosphere, using representative Ar/O_2_ ratios of 10/0, 8/2, and 6/4. For the oxygen-partial-pressure study, Ga_2_O_3_ films were deposited at a fixed annealing temperature of 500 °C with a sputtering time of 20 min. Finally, a 300 nm thick ITO layer was deposited as a transparent top electrode by direct current (DC) magnetron sputtering at 80 W, a pressure of 0.3 Pa, and an Ar flow rate of 7.2 sccm for 30 min. The active region of the device was defined using a photomask with a rectangular geometry of 200 μm × 800 μm. After electrode deposition, the devices were annealed in air for 1 h at three different temperatures. Finally, Ag electrodes were thermally evaporated onto the p^+^-Si substrate to form ohmic contacts. For comparison, single-layer NiO and Ga_2_O_3_ photodetectors were fabricated under the same conditions as the NiO/Ga_2_O_3_ heterojunction devices. The preparation process for gallium oxide thin films in all devices is summarized in Table S1.

### 2.3. Characterization

The surface morphology of the films was characterized by atomic force microscope (AFM, Bruker Multimode 8, Billerica, MA, USA). Crystal structure and phase composition were analyzed by X-ray Diffraction (XRD) using a Panalytical X-ray diffractometer (Almelo, The Netherlands) with Cu K_α_ radiation (λ = 1.5405 Å). X-ray photoelectron spectroscopy (XPS) was employed to determine the elemental composition and chemical states of the film surfaces (analysis depth ~10 nm) [32], using a Thermo Scientific Nexsa system (Waltham, MA, USA) equipped with a monochromatic Al K_α_ X-ray source (1486.6 eV). UV photoelectron spectroscopy (UPS) was performed on the same system using a He excitation source (21.22 eV) to investigate the valence-band and work function [33]. Optical absorption properties were measured by ultraviolet-visible (UV-Vis) spectroscopy using a UV-Vis spectrophotometer (HP 8453E, Agilent Technologies, Santa Clara, CA, USA) coupled with a time-correlated single-photon counting system (C11367-11, Hamamatsu Photonics, Hamamatsu City, Japan). The optoelectronic characteristics of the photodetectors were measured using a semiconductor parameter analyzer (Keysight B1500, Santa Rosa, CA, USA). Optical pattern recognition measurements were carried out by scanning a laser-cut photomask under 254 nm illumination with an XY translation stage and reconstructing the recorded photocurrent signals into a two-dimensional image.

## 3. Results and Discussion

**Figure 2:**
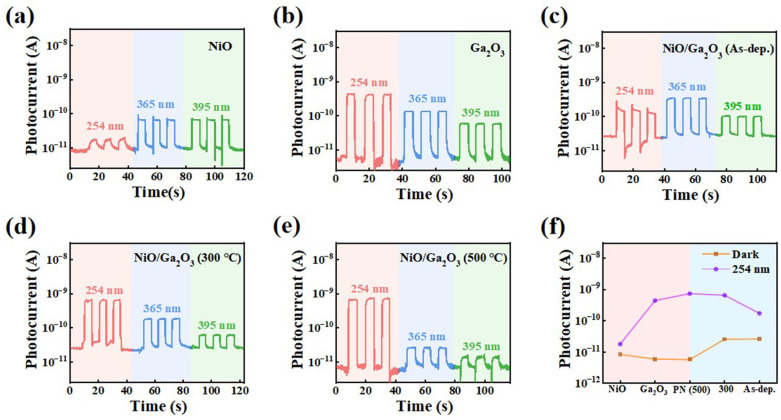
Time-dependent photocurrent responses of (**a**) single-layer NiO film, (**b**) single-layer Ga_2_O_3_ film, and NiO/Ga_2_O_3_ heterojunction devices under different annealing conditions: (**c**) as-deposited, (**d**) annealed at 300 °C, and (**e**) annealed at 500 °C. (**f**) Comparison of the dark current and photocurrent at 254 nm for all devices at zero bias.

To illustrate the design advantages and operating mechanism of the NiO/Ga_2_O_3_ vertical heterojunction, the photo-response evolution from the substrate to the complete device was systematically investigated. Figure 2a,b,e compare the photo-response characteristics of single-layer NiO, single-layer Ga_2_O_3_, and NiO/Ga_2_O_3_ heterojunction photodetectors under 254 nm solar-blind UV and longer-wavelength UV illumination (365 nm and 395 nm) at zero bias. All devices exhibit stable and repeatable photo-switching behavior, while the heterojunction device delivers the strongest response at 254 nm, highlighting the synergistic role of each constituent layer.

As the baseline, the p^+^-Si substrate exhibits pronounced responses to 365 nm and 395 nm illumination but negligible response to 254 nm light (Figure S1), underscoring the necessity of functional layer integration for solar-blind selectivity. Introducing a NiO layer effectively suppresses the substrate-induced long-wavelength photoresponse. After annealing at 500 °C in air, the optimized NiO device exhibits a remarkably reduced dark current at the picoampere level and efficient rejection of long-wavelength illumination (Figure S2), confirming that the NiO layer acts as an effective electrical filter and hole-transport layer. In contrast, single-layer Ga_2_O_3_ devices display strong photoresponse at 254 nm (Figure 2b) but still suffer from insufficient suppression of longer-wavelength interference, demonstrating the limitation of relying on absorption selectivity alone.

By integrating a defect-engineered Ga_2_O_3_ layer with the optimized NiO layer to form a vertical heterojunction (Figure 2c–e), high responsivity and spectral selectivity are simultaneously achieved. The heterojunction preserves the long-wavelength suppression enabled by NiO while retaining the strong deep-UV absorption of Ga_2_O_3_. Furthermore, the built-in electric field at the NiO/Ga_2_O_3_ interface significantly enhances carrier separation under zero bias, resulting in a pronounced photovoltaic-mode photoresponse [34,35,36,37]. The progressive performance enhancement indicates that the device is not a simple functional superposition. Instead, a clear division of labor is established, with NiO acting as an electrical filter and hole-transport layer and Ga_2_O_3_ serving as the solar-blind absorber and electron-transport layer. The cooperative operation enables a self-powered photodetector with both high responsivity and high rejection ratio (Figure 2f).

Post-deposition annealing is known to influence the interfacial quality and optoelectronic performance of oxide heterojunctions. Previous studies have shown that magnetron-sputtered Ga_2_O_3_ films remain amorphous below crystallization temperatures exceeding 600 °C [38], which is consistent with the XRD results of Ga_2_O_3_ films annealed at different temperatures in this work (Figure S3). As shown in Figure 2c–e, increasing the annealing temperature simultaneously enhances the responsivity at 254 nm and the spectral rejection ratio (R_254_/R_365_), with the device annealed at 500 °C exhibiting optimal performance. The enhanced short-wavelength photoresponse observed in Figure 2f indicates that annealing effectively improves heterojunction interface quality and suppresses defect-assisted recombination, thereby promoting efficient deep-UV photovoltaic detection.

**Figure 3:**
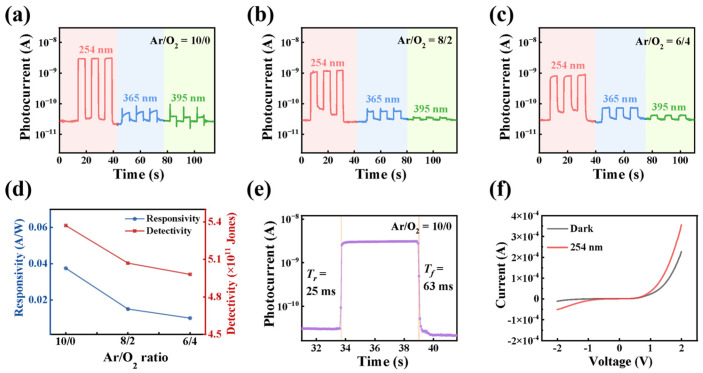
Photodetection performance of NiO/Ga_2_O_3_ heterojunction devices fabricated under different Ar/O_2_ flow ratios: (**a**–**c**) time-dependent photocurrent responses, (**d**) corresponding responsivity and detectivity under 254 nm illumination, (**e**) temporal response characteristics, and (**f**) current-voltage characteristic of the optimal device.

Building on the optimized annealing condition (500 °C), the impact of sputtering atmosphere on Ga_2_O_3_ film properties and NiO/Ga_2_O_3_ heterojunction performance was systematically investigated. Ga_2_O_3_ films and corresponding NiO/Ga_2_O_3_ devices were fabricated under three representative Ar/O_2_ ratios (10/0, 8/2, and 6/4), as summarized in Figure 3. Prior to analyzing device characteristics, the structural nature of the Ga_2_O_3_ films was examined to exclude crystallization-related effects. XRD patterns of all films deposited under different Ar/O_2_ ratios exhibit broad, featureless diffraction humps in the 2θ range of 20–40° (Figure S3), characteristic of amorphous Ga_2_O_3_ [39]. A weak diffraction feature near 35° gradually diminishes with increasing oxygen content, indicating subtle short-range ordering under oxygen-deficient conditions. Importantly, no crystalline phases are detected across the entire oxygen partial pressure range, confirming that subsequent performance variations originate from intrinsic defect modulation rather than phase transitions [40,41].

AFM measurements further reveal systematic changes in surface morphology with varying Ar/O_2_ ratios. As the oxygen proportion increases, the RMS surface roughness decreases from 1.21 nm to 0.75 nm (Figure S4), consistent with a transition from three-dimensional island-like growth to a smoother, layer-like deposition mode [42,43]. Higher oxygen content enhances the oxidation of sputtered species prior to surface incorporation, reduces surface diffusion length, and suppresses coalescence, resulting in denser and more uniform films. Although oxygen partial pressure clearly influences microscopic growth behavior, this morphological evolution alone does not account for the observed device performance trends, as discussed below.

The photovoltaic characteristics of the NiO/Ga_2_O_3_ heterojunction devices were evaluated under zero bias to directly assess the influence of sputtering atmosphere (Figure 3a–d). While the dark current remains nearly unchanged with increasing oxygen content, the photocurrent under 254 nm illumination decreases monotonically as the Ar/O_2_ ratio decreases. Devices fabricated under oxygen-deficient conditions (Ar/O_2_ = 10/0) exhibit the strongest deep-UV photoresponse. In addition, pronounced transient photocurrent peaks are observed at 365 nm and 395 nm for these devices, which can be attributed to rapid carrier trapping and detrapping processes associated with oxygen-vacancy-related defect states. Quantitatively, the responsivity (*R*) and specific detectivity (D*) are defined as [44]:

(1) $$R=\frac{I_{\mathrm{ph}}}{\mathrm{PA}}$$

(2) $$D^{*}=\frac{1}{NEP}=\frac{R\sqrt{A}}{\sqrt{2eI_{d}}}$$

where *I*_ph_ is the photocurrent, *P* is the incident optical power density, *A* is the active area, *NEP* represents noise-equivalent power, *e* is the elementary charge, and *I*_d_ is the dark current. Under optimized deposition conditions (Ar/O_2_ = 10/0), the device achieves a responsivity of 47 mA W^−1^ at 254 nm, a high solar-blind rejection ratio (*R*_254_/*R*_365_) of 4.7 × 10^4^, and a detectivity of 7.52 × 10^11^ Jones under zero bias. Both *R* and D* decrease monotonically with increasing oxygen content, a trend opposite to that of surface roughness evolution (Figure S4), indicating that the device performance is governed primarily by oxygen-vacancy-mediated electronic modulation rather than morphological effects.

Dynamic photoresponse measurements further reveal the operational mechanism of the heterojunction (at zero bias). The fast rise time (*T*_r_ = 25 ms, Figure 3e) reflects rapid carrier generation, while the slower decay (*T*_f_ = 63 ms) is indicative of persistent photoconductivity (PPC). In the Type-II NiO/Ga_2_O_3_ heterojunction, the built-in electric field facilitates efficient spatial separation of photogenerated carriers, causing the recovery process to be dominated by the thermal de-trapping of electrons in Ga_2_O_3_ rather than holes. Based on the standard thermal emission model, *τ* = (1/ν) exp (*E*_a_/k_B_T) (where ν is the attempt-to-escape-frequency, ~10^12^ s^−1^), the measured *T*_f_ corresponds to an activation energy of ~0.65 eV, which aligns well with reported deep-level electron traps (e.g., Oxygen vacancies) in Ga_2_O_3_ [45,46]. This mechanism provides a physically consistent explanation for the observed response time with Ga_2_O_3_ defect physics. The corresponding *I*-*V* characteristics (Figure 3f) demonstrate clear rectifying behavior in the dark and a pronounced photocurrent shift under 254 nm illumination, confirming the formation of a functional *p*-*n* heterojunction. Together, these results establish a direct correlation between sputtering atmosphere, defect concentration, and heterojunction optoelectronic performance.

To elucidate the chemical origins underlying the Ar/O_2_-dependent evolution of Ga_2_O_3_ thin-film properties and device performance, XPS was employed to analyze the gallium and oxygen chemical states. The Ga 3d core-level spectra of Ga_2_O_3_ films deposited under different Ar/O_2_ ratios are shown in Figure S5a–c. Peak deconvolution reveals three characteristic components: a dominant Ga^3+^ peak at 19.8–20.2 eV corresponding to fully coordinated Ga-O bonds in Ga_2_O_3_, a lower binding energy component at ~18.8 eV attributed to reduced Ga species (Ga^+^/Ga^2+^) associated with oxygen-vacancy-related defects, and a minor Ga II component related to surface- or interface-associated gallium species [47]. Quantitative analysis (Figure S5d) shows that increasing the oxygen proportion from 0% to 40% leads to a gradual increase in the Ga^3+^ fraction from 65.8% to 67.9%, indicating a transition from oxygen-deficient Ga_2_O_3−x_ toward a more stoichiometric composition. Meanwhile, the Ga 3d peak position shifts slightly toward lower binding energy with increasing oxygen contents, reflecting enhanced Ga-O coordination and a more stabilized chemical environment. These results demonstrate that adjusting the Ar/O_2_ ratio effectively regulates the gallium oxidation state by controlling oxygen availability during sputtering.

**Figure 4:**
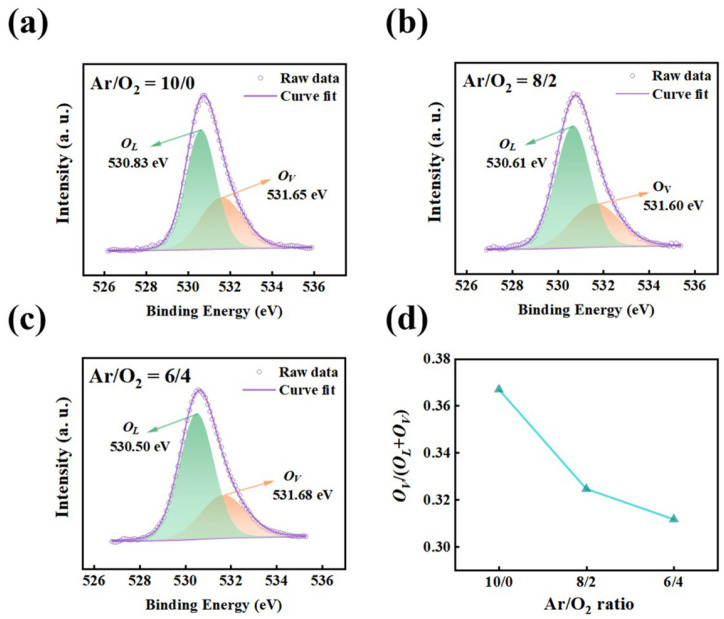
XPS analysis of Ga_2_O_3_ films deposited under different Ar/O_2_ flow ratios: (**a**–**c**) O 1s core-level spectra with peak deconvolution and (**d**) quantified oxygen vacancy fraction as a function of Ar/O_2_ ratio.

To directly probe oxygen-related defects, the O 1s core-level spectra were further analyzed, as shown in Figure 4a–c. The spectra can be deconvoluted into two primary components: a lattice oxygen peak (*O_L_*) at approximately 530.0 eV, corresponding to Ga-O bonds in the Ga_2_O_3_ framework, and a higher binding energy component at 531.0–531.8 eV, which is commonly assigned to oxygen vacancies (*O_V_*) and low-coordination oxygen environment [48]. As summarized in Figure 4d, the relative intensity of the *O_V_* component decreases systematically with increasing oxygen partial pressure, with the oxygen vacancy fraction decreasing from 36.7% to 31.2%. This trend confirms that higher oxygen content during deposition effectively suppresses oxygen vacancy formation. Under oxygen-deficient conditions, preferential sputtering and limited oxygen supply promote vacancy generation, whereas increased oxygen partial pressure compensates sputtering-induced oxygen loss and facilitates the formation of well-coordinated Ga-O bonds. Overall, the Ga 3d and O 1s analyses consistently demonstrate that the Ar/O_2_ ratio provides a reliable means to modulate the oxygen vacancy concentration in amorphous Ga_2_O_3_ films. This controlled defect evolution establishes a clear chemical foundation for understanding the subsequent changes in electronic structure and device performance.

**Figure 5:**
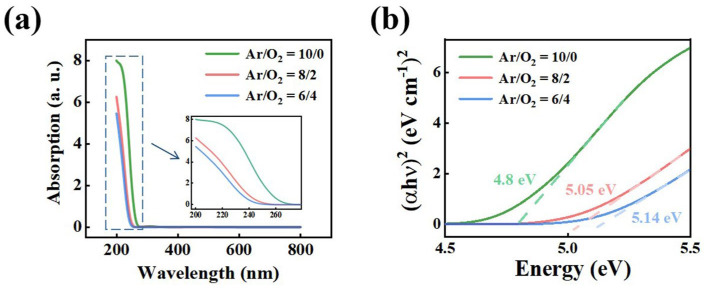
Optical absorption properties of Ga_2_O_3_ films deposited under different Ar/O_2_ flow ratios: (**a**) UV-Vis absorption spectra and (**b**) Tauc plots used for bandgap estimation.

To assess how defect modulation influences the electronic structure of Ga_2_O_3_ films, their optical bandgap (*E*_g_) was characterized using UV-vis absorption spectroscopy. Figure 5a shows the absorption spectra of films deposited under different Ar/O_2_ ratios, all exhibiting strong UV absorption consistent with the wide bandgap nature of Ga_2_O_3_. A systematic blue shift in the absorption edge is observed as the Ar/O_2_ ratio decreases (i.e., with increasing oxygen content), indicating a widening of the bandgap. The absorption data were converted into Tauc plots (Figure 5b) to extract *E*_g_ values. The results reveal that *E*_g_ increases monotonically with higher oxygen partial pressure, rising from 4.8 eV to 5.14 eV. This trend reflects the effect of oxygen vacancies on the electronic structure: higher oxygen content reduces the concentration of vacancy-related defect states, decreasing sub-gap absorption and resulting in a larger effective bandgap. These findings establish a clear link between deposition conditions, defect density, and band structure, providing a foundation for subsequent analysis of band alignment and device performance.

**Figure 6:**
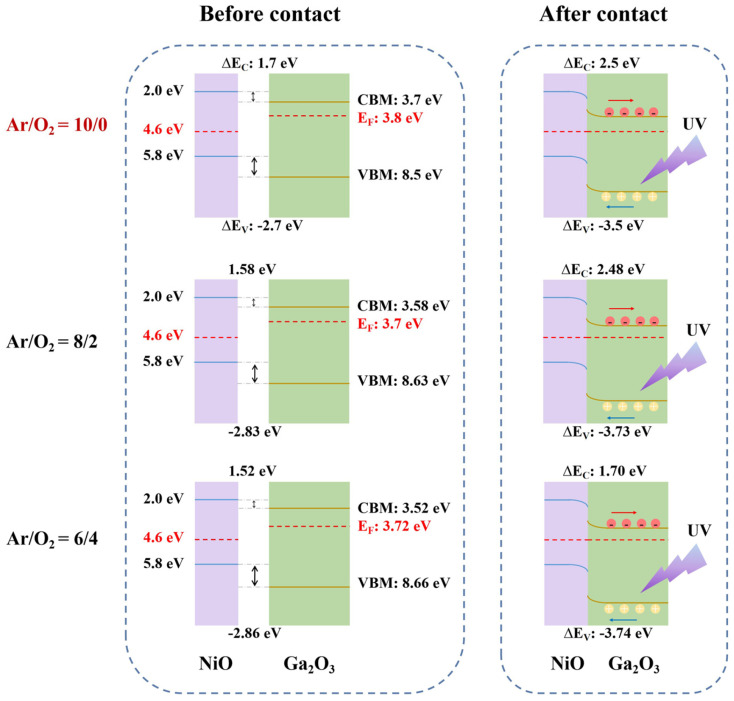
Schematic energy band alignment of NiO/Ga_2_O_3_ heterojunctions for Ga_2_O_3_ films deposited under varying Ar/O_2_ flow ratios, showing band structures before (**left**) and after (**right**) heterojunction formation.

To elucidate how oxygen vacancy modulation influences heterojunction energetics, the electronic structures of Ga_2_O_3_ films prepared under different Ar/O_2_ ratios were systematically analyzed using UPS (Figure S6) combined with UV-vis absorption spectroscopy (Figure 5b, Figure S7) [49]. From these measurements, the valence-band maximum (VBM), the conduction-band minimum (CBM), and the Fermi level (*E*_F_) were determined, allowing schematic band diagrams to be constructed for both isolated Ga_2_O_3_ films and NiO/Ga_2_O_3_ heterojunctions (Figure 6 and Figure S8). The results reveal that the band positions of Ga_2_O_3_ are highly sensitive to the oxygen content. As the Ar/O_2_ ratio decreases from 10/0 to 6/4, the CBM shifts upward from 3.70 eV to 3.52 eV, while the VBM shifts downward from 8.50 eV to 8.66 eV. The energy difference between the conduction band and the Fermi level (*E*_C_ − *E*_F_) increases from 0.1 to 0.20 eV, indicating a reduction in the effective electron concentration, consistent with suppression of shallow donor states associated with oxygen vacancies. In contrast, the absolute Fermi level relative to the vacuum level (*E*_F_ − *E*_vac_) shows a non-monotonic trend (−3.80, −3.70, −3.72 eV), reflecting a competition between shallow donor states (oxygen vacancies) and acceptor-like defects (gallium vacancies). This evolution of *E*_F_ closely tracks the Ga^+^/Ga^2+^ ratio from XPS (Figure S5d), demonstrating that the defect chemistry directly governs band structure modulation.

After forming the NiO/Ga_2_O_3_ heterojunction (Table 1), increasing oxygen content reduces the conduction band offset (Δ*E*_C_) from 2.50 eV to 1.70 eV and increases the valence band offset (|Δ*E*_V_|) from 3.5 eV to 3.74 eV, while the built-in potential (*V*_bi_) varies between 0.80–0.90 eV. Δ*E*_V_ affects hole injection efficiency, Δ*E*_C_ controls electron blocking, and *V*_bi_ sets the driving force for carrier separation. The most favorable configuration for solar-blind photodetection (lowest |Δ*E*_V_|, highest Δ*E*_C_, and strong *V*_bi_) is achieved at Ar/O_2_ = 10/0, which aligns with the observed device performance (Figure 3). These results demonstrate that controlling the Ar/O_2_ ratio allows simultaneous tuning of defect concentrations, including oxygen and gallium vacancies, and the band structure of Ga_2_O_3_ films. This defect-band coupling provides a clear mechanism for understanding how deposition conditions influence heterojunction performance in solar-blind photodetectors.

**Figure 7:**
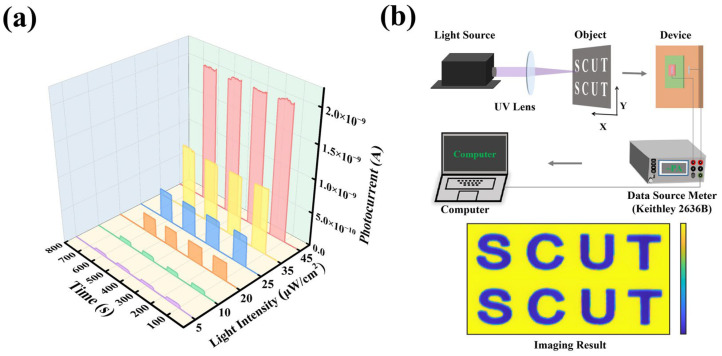
(**a**) Photocurrent response of the optimized NiO/Ga_2_O_3_ heterojunction device under 254 nm illumination, and (**b**) schematic of the solar-blind UV imaging setup with the reconstructed “SCUT” pattern.

To evaluate the operational characteristics of the NiO/Ga_2_O_3_ heterojunction photodetector, its photoresponse under varying illumination conditions was systematically examined. As shown in Figure 7a, the photocurrent increases monotonically with increasing intensity of 254 nm UV illumination, demonstrating a clear power-dependent response. The device exhibits high photosensitivity with stable and reproducible photoresponses under repeated on-off cycling, enabling reliable detection of solar-blind UV light across a wide dynamic range. Compared with previously reported Ga_2_O_3_-based solar-blind photodetectors (Table S2), the NiO/Ga_2_O_3_ heterojunction device demonstrates a well-balanced performance, simultaneously achieving high responsivity, competitive detectivity, and fast response speed under self-powered operation. This performance advantage highlights the effectiveness of the synergistic modulation of the defect-band structure in optimizing both sensitivity and dynamic photoresponse without relying on external bias.

To validate the application feasibility of NiO/Ga_2_O_3_ photodetectors and their stability in repeated detection, optical pattern recognition experiments were further conducted using a solar-blind UV imaging setup. As shown in Figure 7b, a laser-cut photomask with the “SCUT” pattern was illuminated by 254 nm UV light focused by a convex lens and scanned using a computer-controlled XY translation stage, while the detector synchronously recorded the photocurrent. The acquired signals were reconstructed into a two-dimensional image, which exhibits well-defined features with high contrast, confirming effective signal detection and low background interference in the solar-blind UV regime. This result directly reflects the device’s low dark current, high signal-to-noise ratio, and stable photoresponse in the solar-blind UV region. The successful pattern reconstruction confirms that the NiO/Ga_2_O_3_ heterojunction photodetector is capable of real-time, high-contrast solar-blind UV imaging, supporting its potential for practical applications such as UV sensing, security monitoring, and space-based detection systems.

## 4. Conclusions

In this study, a synergistic optimization of the material properties and optoelectronic performance of Ga_2_O_3_ thin films was achieved by systematically controlling the Ar/O_2_ ratio during magnetron sputtering. As the oxygen proportion increased from 0% to 40%, the chemical composition of the films evolved markedly, with the oxygen-vacancy fraction decreasing from 36.7% to 31.2%, demonstrating effective regulation of intrinsic defect states. Although all Ga_2_O_3_ films remained amorphous, the controlled variation in the defect concentration induced pronounced modulation of the electronic structure and heterojunction energetics. In particular, with increasing oxygen content, the Δ*E*_C_ decreases from 2.50 eV to 1.70 eV, the |Δ*E*_V_| increases from 3.5 eV to 3.74 eV, and *V*_bi_ varies from 0.80 eV to 0.90 eV, revealing the strong sensitivity of band alignment to oxygen-vacancy concentration.

Based on the optimized defect configuration and band alignment, the NiO/Ga_2_O_3_ vertical heterojunction device exhibits excellent self-powered solar-blind UV photodetector performance. Under zero bias, the optimized device achieves a responsivity of 47 mA W^−1^ at 254 nm, an ultrahigh wavelength rejection ratio (*R*_254_/*R*_365_) exceeding 10^4^, and fast photoresponse characteristics with a rise time of 25 ms and a fall time of 63 ms. These results confirm the dual role of oxygen vacancies in solar-blind photodetection, simultaneously influencing UV absorption and heterojunction band alignment. More importantly, this work elucidates the critical correlation between process-induced defect density, band alignment, and device performance in Ga_2_O_3_-based solar-blind photodetectors.

## Figures and Tables

**Table 1:** Band energy parameters and band offsets of Ga_2_O_3_ films and NiO/Ga_2_O_3_ heterojunctions prepared at different Ar/O_2_ ratios.

Ar/O_2_ Ratio	Before Contact (Ga_2_O_3_)			After Contact (NiO/Ga_2_O_3_)		
	CBM (eV)	VBM (eV)	*E_F_* (eV)	Δ*E*_C_	|Δ*E*_V_|	*V*_bi_
10/0	3.70	8.50	3.80	2.50	3.50	0.80
8/2	3.58	8.63	3.70	2.48	3.73	0.90
6/4	3.52	8.66	3.72	1.70	3.74	0.88

## Data Availability

The original contributions presented in this study are included in the article/Supplementary Materials. Further inquiries can be directed to the corresponding authors.

## Supplementary Materials

The following supporting information can be downloaded at: https://www.mdpi.com/article/10.3390/ma19030530/s1, Table S1: Deposition and annealing conditions of Ga_2_O_3_ for all samples; Figure S1: Time-dependent photocurrent responses of p+-Si; Figure S2: Time-dependent photocurrent responses of a single-layer NiO film (a) as-deposited, (b) 300 °C-annealed, and (c) 500 °C-annealed, with (d) comparison of dark current for all devices; Figure S3: XRD analysis of Ga_2_O_3_ films deposited under different Ar/O_2_ flow ratios; Figure S4: AFM profiles of Ga_2_O_3_ films: (a–c) deposited under different Ar/O_2_ flow ratios and (d) comparison of RMS for all devices; Figure S5: XPS analysis of Ga_2_O_3_ films deposited under different Ar/O_2_ flow ratios: (a–c) Ga 3d core-level spectra with deconvolution, and (d) corresponding Ga_I_ ratio and Ga_III_ ratio as a function of Ar/O_2_ ratio; Figure S6: UPS plots of Ga_2_O_3_ films: (a–c) deposited under different Ar/O_2_ flow ratios and (d) comparison of VBM for all devices; Figure S7: Optical absorption properties of NiO films: (a) absorption spectra and (b) Tauc plots for energy bandgap estimation; Figure S8: UPS analysis of NiO films; Table S2: Performance comparison of Ga_2_O_3_-based UV photodetectors. References [50,51,52,53,54,55,56,57,58,59,60] are cited in the Supplementary Materials.
