# Supplementary material for: NiO/Ga2O3 Heterojunction with Tunable Oxygen Vacancies for Efficient Self-Powered Solar-Blind UV Detection
Source: Materials (Basel). 2026 Jan 29;19(3):530. doi: 10.3390/ma19030530 (PMC12898588; doi:10.3390/ma19030530)
Supplement: Supplementary file 1 [file materials-19-00530-s001.zip › materials-4102767-supplementary.pdf]

Supporting Information

NiO/Ga_2O_3 Heterojunction with Tunable Oxygen Vacancies for Efficient Self-Powered Solar-Blind UV Detection

Luyu Liu,[1,2] Kangxin Shen,[1,2] Humin Su,[1,2] Jintao Xu,[1,2] Jiajun Lin,[1,2] Yaping Li,[3,*] Shuguang Zhang,[1,2,*] Linfeng Lan,[1,2,*] and Junbiao Peng[1,2]

[1] *State Key Laboratory of Luminescent Materials and Devices, South China University of Technology, Guangzhou 510640, China*

[2] *School of Materials Science and Engineering, South China University of Technology, Guangzhou 510640, China*

[3] *Guangdong Provincial Key Laboratory of In-Memory Computing Chips School of Electronic and Computer Engineering, Peking University, Shenzhen 518055, China*

[*] *Author to whom correspondence should be addressed.*

E-mail: liyaping_415@163.com; mssgzhang@scut.edu.cn; lanlinfeng@scut.edu.cn

Table S1. Deposition and annealing conditions of Ga_2O_3 for all samples

**Samples**	**Sputtering power (W)**	**Working pressure (Pa)**	**Ar/O_2 ratio**	**Annealing temperature (°C)**	**Sputtering time (min)**
Fig. 2b	100 (RF)	0.5	10/0	500	20
Fig. 2c	100 (RF)	0.5	10/0	As-deposited	20
Fig. 2d	100 (RF)	0.5	10/0	300	20
Fig. 2e	100 (RF)	0.5	10/0	500	20
Fig. 3a	100 (RF)	0.5	10/0	500	20
Fig. 3b	100 (RF)	0.5	8/2	500	20
Fig. 3c	100 (RF)	0.5	6/4	500	20

Notes: Although Figures 2e and 3a involve nominally identical preparation conditions, minor variations in the *I–T* curves may arise from differences in testing conditions or between sample batches. However, the dark-to-light current ratios and solar-blind rejection ratios remain consistent, confirming the reliability and reproducibility of the device performance.

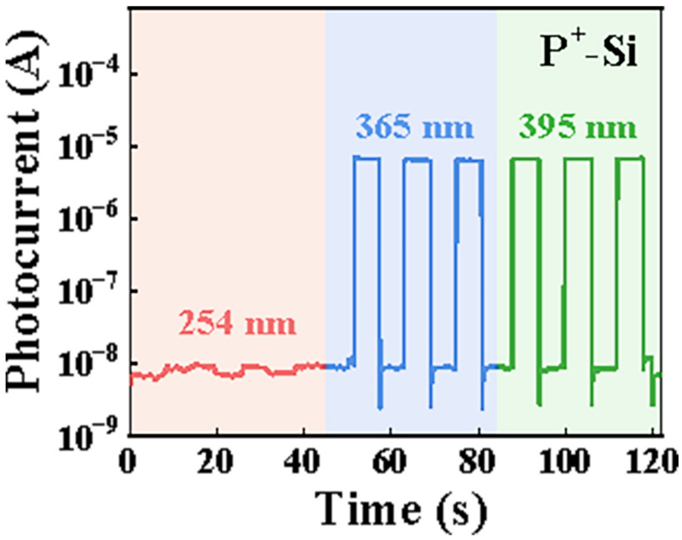

Figure S1. Time-dependent photocurrent responses of p^+-Si

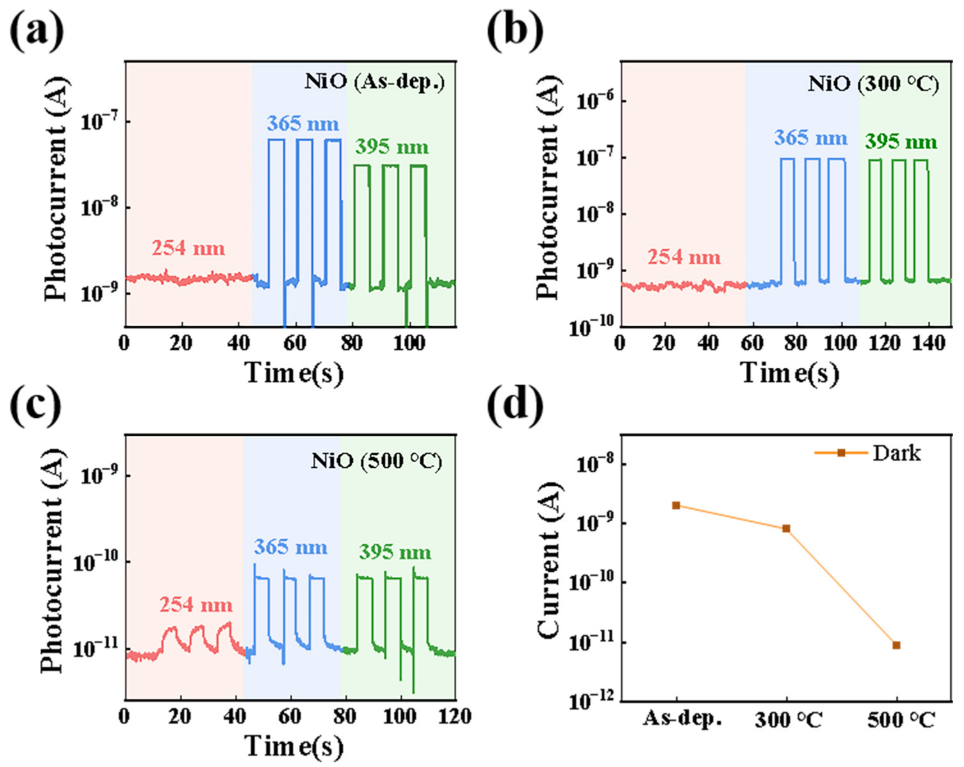

Figure S2. Time-dependent photocurrent responses of a single-layer NiO film (a) as-deposited, (b) 300 °C-annealed, and (c) 500 °C-annealed, with (d) comparison of dark current for all devices.

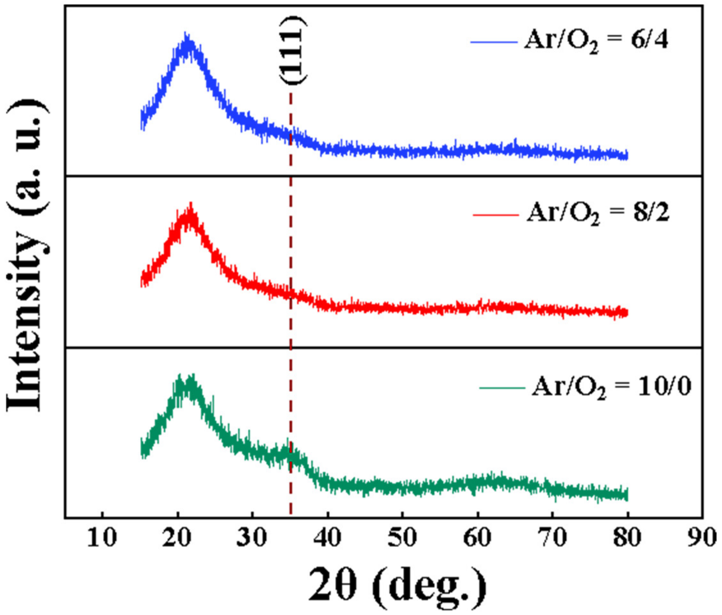

Figure S3. XRD analysis of Ga_2O_3 films deposited under different Ar/O_2 flow ratios

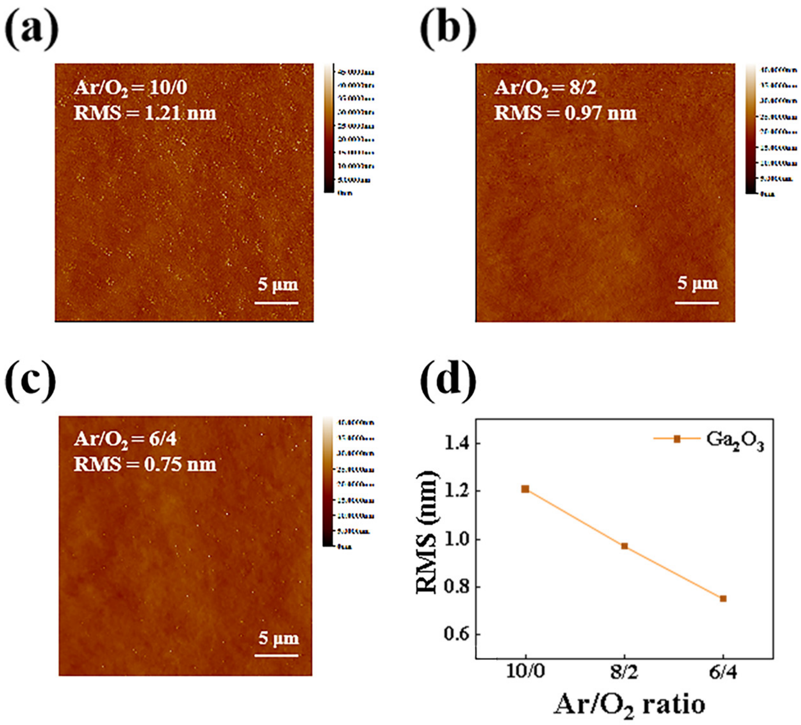

Figure S4. AFM profiles of Ga_2O_3 films: (a-c) deposited under different Ar/O_2 flow ratios and (d) comparison of RMS for all devices.

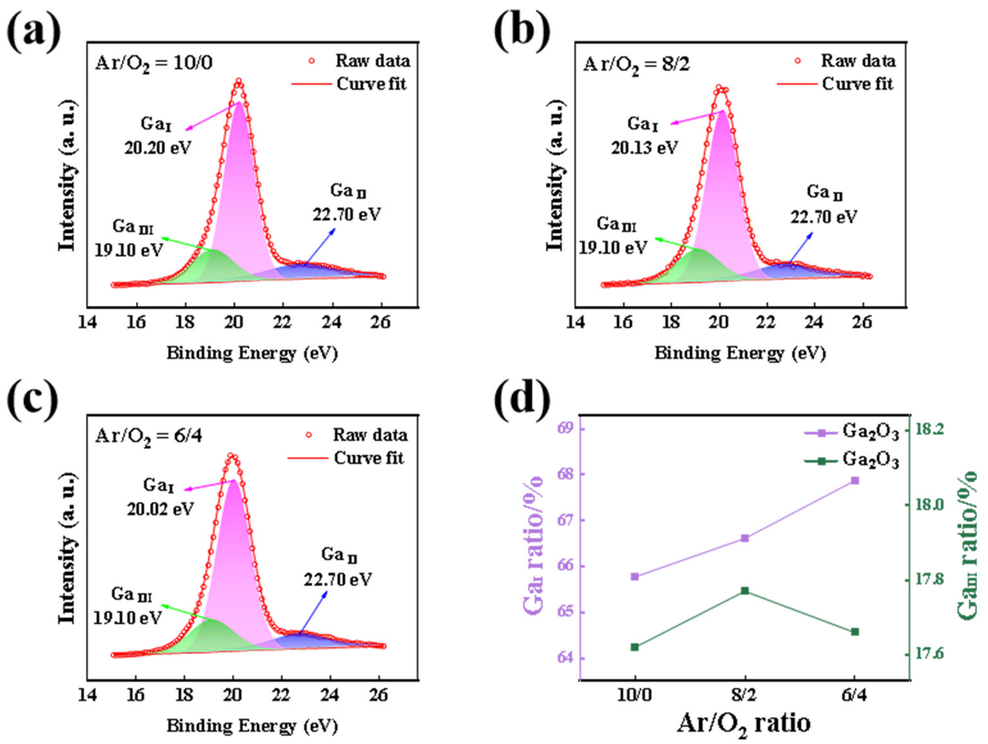

Figure S5. XPS analysis of Ga_2O_3 films deposited under different Ar/O_2 flow ratios: (a-c) Ga 3d core-level spectra with deconvolution, and (d) corresponding Ga_I ratio and Ga_{III} ratio as a function of Ar/O_2 ratio.

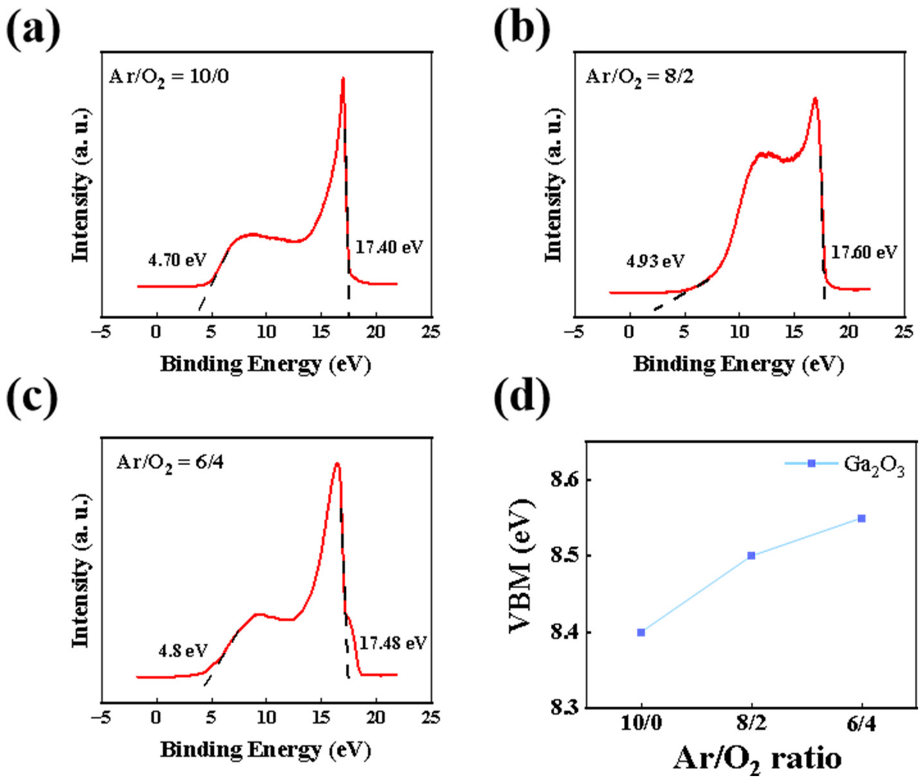

Figure S6. UPS plots of Ga_2O_3 films: (a-c) deposited under different Ar/O_2 flow ratios and (d) comparison of VBM for all devices.

The UPS spectra were measured using He I excitation (21.2 eV). The valance band maximum (VBM) was calculated as:

$$\text{VBM} = 21.2\ \text{eV} - (E_{\text{cutoff}} - E_{\text{VB}})$$

where E_{cutoff} is the high-binding-energy cutoff of the UPS spectrum, and E_{VB} is the valance band onset (low-binding-energy cutoff). The Fermi level (E_{F}) was estimated using:

$$E_{\text{F}} = 21.2\ \text{eV} - E_{\text{cutoff}}$$

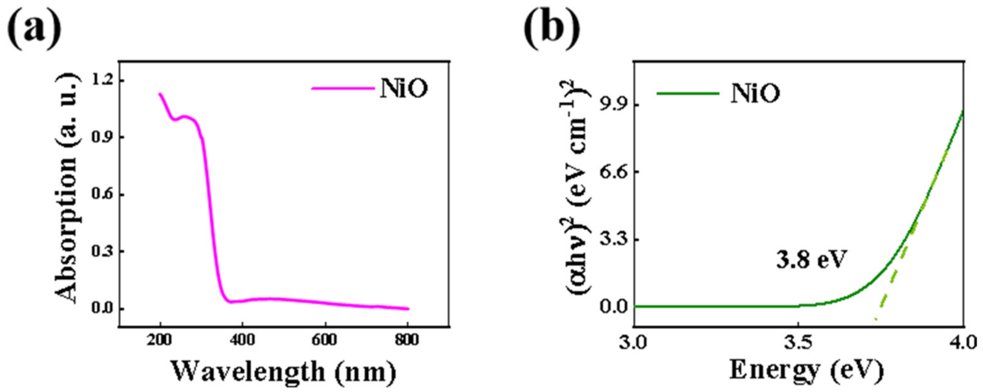

Figure S7. Optical absorption properties of NiO films: (a) absorption spectra and (b) Tauc plots for energy bandgap estimation

The bandgap of NiO was determined from UV-Vis absorption spectra using the Tauc plot method. Specifically, $(\alpha h\nu)^2$ was plotted versus hν, the linear portion of the absorption edge was fitted, and the intercept at $(\alpha h\nu)^2=0$ yields E_g.

.

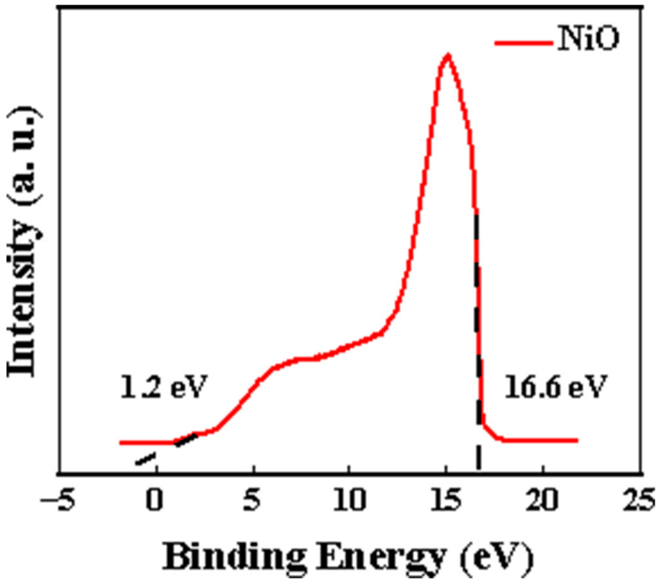

Figure S8. UPS analysis of NiO films

Table S2. Performance comparison of Ga_2O_3-based UV photodetectors

Year	Structures	Responsivity (mA/W)	Detectivity (Jones)	Rise/Fall Time	Spectral Selectivity	Dark Current (A)
2020[1]	β-Ga_2O_3/NiO	4.27	4.2×10^9	4.6/7.6 ms	—	1.09×10^{-9} (-15 V)
2025[2]	NiO/ HR-ε-Ga_2O_3/ LR-ε- Ga_2O_3	160	3.7×10^{12}	38/67 ms	UV/Vis=1.5×10^3	9.9×10^{-9} (-2 V)
2021[3]	$CuMO_2$/ β- Ga_2O_3	0.025 ($CuGaO_2$) 0.12 ($CuCrO_2$)	9×10^{10} 4.6×10^{11}	260/140 ms 350/60 ms	$R_{254}/R_{365} = 2\times10^5$, 2.8×10^4	5.1×10^{-14}, 6.1×10^{-14}
2022[4]	Ag_2O/β-Ga_2O_3	25.65	6.10×10^{11}	108/80 ms	$R_{254}/R_{365} = 2.47\times10^3$	6.55×10^{-11}
2023[5]	Ga_2O_3/ZnO:V	95.53	1.18×10^{12}	6.2/0.43 ms	$R_{260}/R_{400} = 10^4$	1.08×10^{-10}
2022[6]	Ga_2O_3 NRs	3.87	—	0.23/0.15 s	—	—
2023[7]	CuI/Ga_2O_3	1.44	5.94×10^{11}	420/16 ms	—	$10^{-12} \sim 10^{-13}$
2023[8]	β-Ga_2O_3/NiO/ β-Ga_2O_3	10	—	~ μs	—	$\sim1\times10^{-10}$
2025[9]	p-NiO/ n-κ- Ga_2O_3	0.325	1.2×10^{10}	< 0.8 s	—	1×10^{-10}
2023[10]	Ga_2O_3/GaN	44.98 (0 V) 125.73 (2 V)	5.3×10^{11} (0 V) 7×10^{11} (2 V)	383/96 ms	—	$\sim10^{-10}$ (0 V)
2024[11]	n-Si/n- Ga_2O_3/ p-Li:NiO	0.18	1.57×10^9	132/148 ms	$R_{230}/R_{365} = 27$	—
This work	NiO/Ga_2O_3	47	3×10^{11}	25/63 ms	$R_{254}/R_{365} = 4.7\times10^4$	3×10^{-11}

References:

1. Yu, J.G.; Yu, M.; Wang, Z.; Yuan, L.; Huang, Y.; Zhang, L.C.; Zhang, Y.M.; Jia, R.X. Improved Photoresponse Performance of Self-Powered β-Ga_2O_3/NiO Heterojunction UV Photodetector by Surface Plasmonic Effect of Pt Nanoparticles. *IEEE Trans. Electron Devices* **2020**, *67*, 3199-3204.
2. Zhang, X.L.; Liu, N.T.; Lin, H.B.; Han, D.Y.; Zhang, W.R.; Ye, J.C. Interface-Engineered High-Performance Self-Powered Solar-Blind Photodetector Based on NiO/ε-Ga_2O_3 Heterojunctions. *ACS Appl. Mater. Interfaces* **2025**, *17*, 17117-17126.
3. Wu, C.; Qiu, L.L.; Li, S.; Guo, D.Y.; Li, P.G.; Wang, S.L.; Du, P.F.; Chen, Z.W.; Liu, A.P.; Wang, X.H.; Wu, H.P.; Wu, F.M.; Tang, W.H. High sensitive and stable self-powered solar-blind photodetector based on solution-processed all inorganic $CuMO_2$/Ga_2O_3 pn heterojunction. *Mater. Today Phys.* **2021**, *17*, 100335.
4. Park, S.; Park, T.; Park, J.H.; Min, J.Y.; Jung, Y.; Kyoung, S.; Kang, T.Y.; Kim, K.H.; Rim, Y.S.; Hong, J. Ag_2O/β-Ga_2O_3 Heterojunction-Based Self-Powered Solar Blind Photodetector with High Responsivity and Stability. *ACS Appl. Mater. Interfaces* **2022**, *14*, 2648-2658.
5. Wang, H.B.; Ma, J.A.; Chen, H.; Wang, L.P.; Li, P.; Liu, Y.C. Ferroelectricity enhanced self-powered solar-blind UV photodetector based on Ga_2O_3/ZnO:V heterojunction. *Mater. Today Phys.* **2023**, *30*, 100929.
6. Fan, M.M.; Xu, K.L.; Li, X.Y.; He, G.H.; Cao, L. Self-powered solar-blind UV/visible dual-band photodetection based on a solid-state PEDOT:PSS/α-Ga_2O_3 nanorod array/FTO photodetector. *J. Mater. Chem. C* **2021**, *9*, 16459-16467.
7. Liu, Y.Z.; Shen, L.Y.; Pan, X.H.; Zhang, T.; Wu, H.S.; Wang, N.; Wang, P.; Wang, F.Z.; Ye, Z.Z. Self-powered solar-blind deep-UV photodetector based on CuI/Ga_2O_3 heterojunction with high sensitivity. *Sens. Actuators, A* **2023**, *349*, 114068.
8. Nakagomi, S. Ultraviolet Photodetector Based on a Beta-Gallium Oxide/Nickel Oxide/Beta-Gallium Oxide Heterojunction Structure. *Sensors* **2023**, *23*, 8332.
9. Moumen, A.; Kalvani, P.R.; Mattei, F.; Foti, G.; Parisini, A.; Mosca, R.; Pavesi, M.; Bosi, M.; Seravalli, L.; Mezzadri, F.; Baraldi, A.; Mazzolini, P.; Vantaggio, S.; Bosio, A.; Fornari, R. Self-powered NiO/κ-Ga_2O_3 heterojunction photodiode for fast broadband ultraviolet (UV) radiation detection. *Opt. Mater.* **2025**, *165*, 117125.
10. Feng, S.Y.; Liu, Z.T.; Feng, L.Z.; Wang, J.C.; Xu, H.N.; Deng, L.J.; Zhou, O.X.; Jiang, X.; Liu, B.D.; Zhang, X.L. High-performance self-powered ultraviolet photodetector based on Ga_2O_3/GaN heterostructure for optical imaging. *J. Alloys Compd.* **2023**, *945*, 169274.
11. Zhang, X.; Yue, Z.; Zhao, E.Q.; Wei, S.K.; Jiao, C.F.; Xin, M.B.; Wang, K.Y.; Zhai, R.F.; Ye, W.X.; Wang, H.; Zhao, Y. Improved photoresponse performance of self-powered solar-blind UV photodetectors based on n-Si/n-Ga_2O_3/p-Li:NiO dual-junction. *Appl. Phys. Lett.* **2024**, *125*, 233501.
